# Novel immunomodulatory drugs and neo-substrates

**DOI:** 10.1186/s40364-020-0182-y

**Published:** 2020-01-09

**Authors:** Shaobing Gao, Shichao Wang, Yongping Song

**Affiliations:** 1 The Affiliated Cancer Hospital of Zhengzhou University, Henan Cancer Hospital, 127 Dongming Road, Zhengzhou, 450008 China; 2 The Fifth Affiliated Hospital of Zhengzhou University, No. 3 Kangfu Front Street, Zhengzhou, 450052 China

**Keywords:** Immunomodulatory drugs, CRL4^CRBN^ E3 ligase, CC-122, CC-220, PROTACs

## Abstract

Thalidomide, lenalidomide and pomalidomide are immunomodulatory drugs (IMiDs) effective in the treatment of multiple myeloma, myelodysplastic syndrome (MDS) with deletion of chromosome 5q and other hematological malignancies. Recent studies showed that IMiDs bind to CRBN, a substrate receptor of CRL4 E3 ligase, to induce the ubiquitination and degradation of IKZF1 and IKZF3 in multiple myeloma cells, contributing to their anti-myeloma activity. Similarly, lenalidomide exerts therapeutic efficacy via inducing ubiquitination and degradation of CK1α in MDS with deletion of chromosome 5q. Recently, novel thalidomide analogs have been designed for better clinical efficacy, including CC-122, CC-220 and CC-885. Moreover, a number of neo-substrates of IMiDs have been discovered. Proteolysis-targeting chimeras (PROTACs) as a class of bi-functional molecules are increasingly used as a strategy to target otherwise intractable cellular protein. PROTACs appear to have broad implications for novel therapeutics. In this review, we summarized new generation of immunomodulatory compounds, their potential neo-substrates, and new strategies for the design of novel PROTAC drugs.

## Background

Thalidomide was notorious for the teratogenic effects leading to congenital malformations in phocomelia infants [1–5]. Thalidomide and its derivatives can modulate functions of T cells and NK cells by inducing the production of cytokines, including IL-2 (interleukin-2) and interferon γ [6–9]. Thus, thalidomide and its analogs, including lenalidomide and pomalidomide, are called immunomodulatory drugs (IMiDs). In addition, IMiDs have antiangiogenic activity [10, 11]. IMiDs are widely used in combination with proteasome inhibitors, steroids, and monoclonal antibodies and playing a pivotal role in the treatment of multiple myeloma (MM) [12–18]. Lenalidomide also showed activities in a number of hematological malignancies, including myelodysplastic syndrome (MDS) with deletion of chromosome 5q (del(5q)) [19–21], mantle cell lymphoma (MCL) [22–27] and chronic lymphocytic leukemia (CLL) [28–31].

Although IMiDs have been approved for the treatment of several hematological malignancies, the molecular mechanism remained unclear at that time. Lately, the primary cellular target of thalidomide was identified to be CRBN, a substrate receptor of Cullin-RING Ligase 4 (CRL4) [32]. IMiDs target CUL4-RBX1-DDB1-CRBN (CRL4^CRBN^) E3 ligase to induce the ubiquitination and proteasomal degradation of Ikaros family zinc finger proteins, Ikaros (IKZF1) and Aiolos (IKZF3) which are the lymphoid transcription factors essential for myeloma cell survival [33–35]. Similarly, lenalidomide induces the ubiquitination and degradation of CK1α, leading to the death of del(5q) MDS cells [36].

**Fig. 1:**
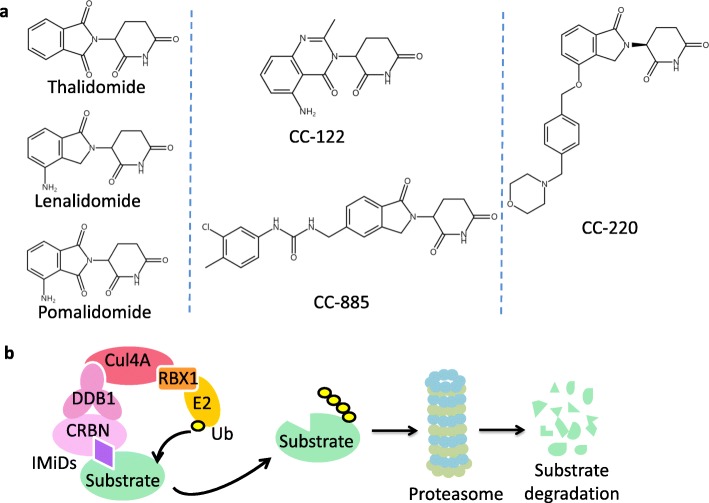
Chemical structure and mechanism of action of IMiDs. **a** Chemical structure of thalidomide, lenalidomide, pomalidomide, CC-122, CC-885 and CC-220. **b** IMiDs (purple rhombuses) bind to CRBN, a substrate receptor of CRL4 E3 ligase, to recruit substrates for ubiquitination and proteasomal degradation. Ub, ubiquitin

Recently, novel thalidomide analogs have been developed, including CC-122 (avadomide), CC-220 (iberdomide) and CC-885 [36–39] (Fig. 1). These novel CRBN modulators are in active clinical trials. Moreover, studies have shown that IMiDs repurpose CRL4^CRBN^ E3 ligase to ubiquitinate and degrade a series of cellular proteins [40, 41].

In this review, we summarized recent advances on new generation of IMiDs, their neo-substrates and the implication for novel therapeutics.

## New generation of immunomodulatory drugs

IMiDs contain a conserved glutarimide ring and a variable phthaloyl ring. The glutarimide ring interacts with a conserved hydrophobic pocket of CRBN. The phthaloyl ring, together with CRBN, forms binding interface for neo-substrates [42–44]. Hence, modifications on the variable phthaloyl ring may lead to new generation of IMiDs.

### CC-122 (avadomide)

CC-122 is a novel immunomodulatory compound containing the conserved glutarimide for CRBN binding. As a derivative of thalidomide, CC-122 has pluripotent activities, including antitumor and modulation of immune cells [37, 45]. CC-122 binds CRL4^CRBN^ E3 ligase to induce the degradation of IKZF1 and IKZF3 in MM cells, diffuse large B-cell lymphoma (DLBCL) cells and xenograft mouse models established from DLBCL cells [36, 37]. The degradation of IKZF1 and IKZF3 results in derepression of IFN-regulated genes, leading to apoptosis of several DLBCL cell lines and inhibition of tumor growth in xenograft mouse models [37]. In addition, CC-122 costimulates T cells and induces the production of IL-2 [37].

Based on the above notable antitumor activities and modulations on immune cells, CC-122 has entered clinical trials for a number of diseases, including Non-Hodgkin’s lymphoma (NHL), MM and CLL/SLL (Table 1). Recently, a multicenter, open-label, and dose-escalation phase 1 clinical trial (NCT01421524) has been conducted to evaluate the safety, tolerability, pharmacokinetics and preliminary efficacy of CC-122 in patients with MM, NHL and advanced solid tumors. In the latest report, 34 patients with NHL, MM or advanced solid tumors were enrolled [46]. These patients had a median age of 57 years and had received a median of 3.5 prior anticancer therapies. In the part A portion of this trial, patients received CC-122 at an increasing dose of 0.5 to 3.5 mg orally once daily on a 28-day schedule. The median duration of CC-122 treatment was 58 days. Fatigue (44%), neutropenia (29%) and diarrhea (15%) were the most common treatment-emergent adverse events (TEAEs). The non-tolerated dose (NTD) was 3.5 mg and maximum tolerated dose (MTD) was 3.0 mg. One of five patients with NHL had a complete response (CR) and two of them achieved partial responses. This study confirmed that CC-122 induces the degradation of IKZF3 protein in a dose-dependent manner in B and T cells from peripheral blood. Moreover, B-cell numbers in peripheral blood were reduced after 15-day administration of CC-122. To summarize, CC-122 monotherapy showed acceptable safety and encouraging pharmacokinetics in patients with MM, NHL and solid tumors [46].

**Table 1:** Clinical trials of CC-122 in cancer

Phase	Conditions	Interventions	NCT ID
1	MM, NHL, solid tumors	CC-122	NCT01421524
1	DLBCL, iNHL	CC-122, obinutuzumab	NCT02417285
1	DLBCL, FL	CC-122, rituximab, CC-223, CC-292	NCT02031419
1, 2	DLBCL	CC-122, R-CHOP	NCT03283202
1	NHL	CC-122	NCT02509039
1, 2	CLL, SLL	CC-122, ibrutinib, obinutuzumab	NCT02406742
1, 2	HCC	CC-122, nivolumab	NCT02859324
2	Melanoma	CC-122, nivolumab	NCT03834623

*Abbreviation*: *CLL* Chronic lymphocytic leukemia, *DLBCL* Diffuse large B-cell lymphoma, *FL* Follicular lymphoma, *HCC* Hepatocellular carcinoma, *MM* Multiple myeloma, *NHL* Non-Hodgkin’s lymphoma, *iNHL* Indolent NHL, *R-CHOP* (Rituximab, cyclophosphamide, doxorubicin, vincristine, prednisone), *SLL* Small lymphocytic lymphoma

A multi-center, open-label, and dose escalation/expansion phase 1 clinical trial (NCT02417285) is ongoing to test the safety, tolerability and preliminary efficacy of CC-122 in combination with obinutuzumab in NHL. According to the interim result, 58 patients were enrolled, including 38 with relapsed or refractory (R/R) follicular lymphoma (FL), 19 with R/R DLBCL and 1 with R/R marginal zone lymphoma [47]. These patients received increasing doses of CC-122 for 5 days per week (5/7 days) in each 28-day cycle in combination with obinutuzumab at a dose of 1000 mg on days 2, 8, and 15 of cycle 1, and day 1 of cycles 2 to 8. Among the 38 patients with R/R FL, the most common TEAEs were neutropenia (66%), pyrexia (29%) and thrombocytopenia (29%). The overall response rate (ORR) was 68% and 16 out of these 38 patients (42%) achieved a CR. CC-122 in combination with obinutuzumab was well-tolerated and showed promising efficacy in patients with R/R FL [47].

In another ongoing multi-center and open-label phase 1 clinical trial (NCT02031419), combinations of CC-122, CC-223, CC-292 and rituximab was given in patients with R/R DLBCL or FL. From the interim result of the arm D of this study, 37 patients with R/R FL received CC-122 at a dose of 2 mg or 3 mg for 5/7 days and intravenous rituximab at a dose of 375 mg/m^2^ in each 28-day cycle [48]. Neutropenia (46%) and anemia (24%) were the most common TEAEs. The ORR was 65% and 8 patients (22%) achieved a CR. Thus, CC-122 in combination with rituximab was well-tolerated and showed promising clinical activity in patients with R/R FL [48].

A phase 1/2 clinical trial (NCT03283202) will evaluate the safety and preliminary efficacy of CC-122 combined with R-CHOP regimen for newly-diagnosed DLBCL patients with poor risk factor (Table 1). Therefore, CC-122 has shown clinical potential for the treatment of MM and NHL.

### CC-220 (iberdomide)

CC-220 is a new analog of thalidomide developed for the treatment of relapsed/refractory MM (RRMM) and systemic lupus erythematosus (SLE). CC-220 has improved efficacy to degrade IKZF1 and IKZF3 through tighter binding to the CRL4^CRBN^ E3 ligase [38].

Recently, a double-blinded, placebo-controlled, single dose-escalation phase 1 study (NCT01733875) has been carried out in healthy volunteers to evaluate safety, pharmacokinetics and pharmacodynamics of CC-220. In the latest report, 56 healthy volunteers were enrolled and randomized into 7 cohorts [49]. In each cohort, six subjects took a single dose of 0.03 to 6 mg CC-220 and two subjects received placebo orally. In this study, no severe TEAEs were reported. CC-220 was well tolerated when taken at a single dose of 6 mg orally in these healthy volunteers. Consistently, CC-220 administration causes the degradation of IKZF1 and IKZF3 in B cells, T cells and monocytes. In addition, CC-220 inhibited the production of anti-dsDNA and anti-phospholipid autoantibodies in cultured peripheral blood mononuclear cells (PBMCs) from SLE patients [49]. Thus, this study demonstrated the tolerated safety and pharmacodynamic activity of CC-220, indicating its promising clinical development for SLE. Soon afterwards, two randomized, placebo-controlled, double-blinded, phase 2 studies (NCT02185040, NCT03161483) in SLE patients were designed to study the safety, tolerability, pharmacokinetics and pharmacodynamics of CC-220 in SLE.

At this time, a multicenter, open-label, and dose-escalation phase 1/2 study (NCT02773030) in RRMM is ongoing to evaluate the safety, tolerability, pharmacokinetics and preliminary efficacy of CC-220 when administered as monotherapy, and in combination with dexamethasone, with or without daratumumab or bortezomib. According to the preclinical studies, CC-220 combined with bortezomib induced deep IKZF1 and IKZF3 degradation at clinically relevant concentrations and showed synergistically antiproliferative effects in MM cell lines, which could be further enhanced by dexamethasone. In addition, CC-220 in combination with bortezomib induced deeper apoptosis than combinations of any other clinically approved IMiDs with bortezomib in MM cell lines. CC-220 also synergistically enhanced the anti-myeloma activity of daratumumab in complement-dependent cytotoxicity assays [50]. From the interim results, 69 patients with RRMM received CC-220 at an increasing dose of 0.3 to 1.3 mg on days 1–21 plus dexamethasone at a dose of 20 mg or 40 mg on days 1, 8,15 and 22 in each 28-day cycle [51]. The ORR was 29% and clinical benefit rate was 45%. The MTD has not been reached. Combination of CC-220 and dexamethasone showed favorable tolerability with grade 3–4 neutropenia (29%), infections (25%), and thrombocytopenia (12%) [51]. These preclinical and clinical data suggest the promising clinical application of CC-220 in combination with bortezomib, dexamethasone and daratumumab for MM treatment.

In general, current clinical trials on CC-220 mostly focus on its potential to treat SLE and MM (Table 2).

**Table 2:** Clinical trials of CC-220

Phase	Conditions	Interventions	NCT ID
1	Healthy volunteers	CC-220, placebo	NCT01733875
2	SLE	CC-220, placebo	NCT03161483
2	SLE	CC-220, placebo	NCT02185040
1,2	MM	CC-220, DEX, Dara, BTZ	NCT02773030
1	Healthy volunteers	CC-220	NCT03135509
1	Healthy volunteers	CC-220, radiation	NCT03294603
1	Hepatic impairment, Healthy volunteers	CC-220	NCT03824678

*Abbreviation*: *SLE* Systemic lupus erythematosus, *DEX* Dexamethasone, *Dara* Daratumumab, *BTZ* Bortezomib

### CC-885

CC-885 is a new CRBN modulator with a strong anti-proliferation activity in a broad set of tumor cell lines [39]. CC-885 can induce CRL4^CRBN^-dependent degradation of IKZF1 and the translation termination factor GSPT1, while neither lenalidomide nor pomalidomide can trigger the depletion of GSPT1, suggesting different substrate spectrum of CC-885 from lenalidomide or pomalidomide. Moreover, CC-885 showed sub-nanomolar potency against patient-derived acute myeloid leukemia (AML) cells, though lenalidomide and pomalidomide do not have significant activity in AML [39]. CC-885 may thus have a potential for AML therapy different from other IMiDs. As CC-885 can induce the degradation of GSPT1 while other IMiDs do not, it may have extra toxicity from other IMiDs. More studies on the activity and toxicity effects of CC-885 are required.

## Potential neo-substrates of immunomodulatory drugs

Structural studies have revealed that neo-substrates of IMiDs-CRL4^CRBN^ complex, including IKZF1, IKZF3, CK1α, ZFP91, share a common structural motif containing a key glycine [42–44, 52]. Hence, cellular proteins which contain this structural feature may be targeted and degraded by IMiDs.

In a recent study, a mass spectrometry-based workflow was established to detect IMiDs-induced target degradation in Kelly, SK-N-DZ, human embryonic stem cells and MM1S cells [40]. These cells were treated with pomalidomide, lenalidomide, thalidomide or DMSO as a control respectively, and protein abundance was measured by multiplexed mass spectrometry. Comprehensive proteomics analysis identified several potential neo-substrates of IMiDs, including ZNF653, ZNF827, ZNF692, RNF166, FAM83F, RAB28, DTWD1, GZF1, ZBTB39, and ZNF98 [40].

**Fig. 2:**
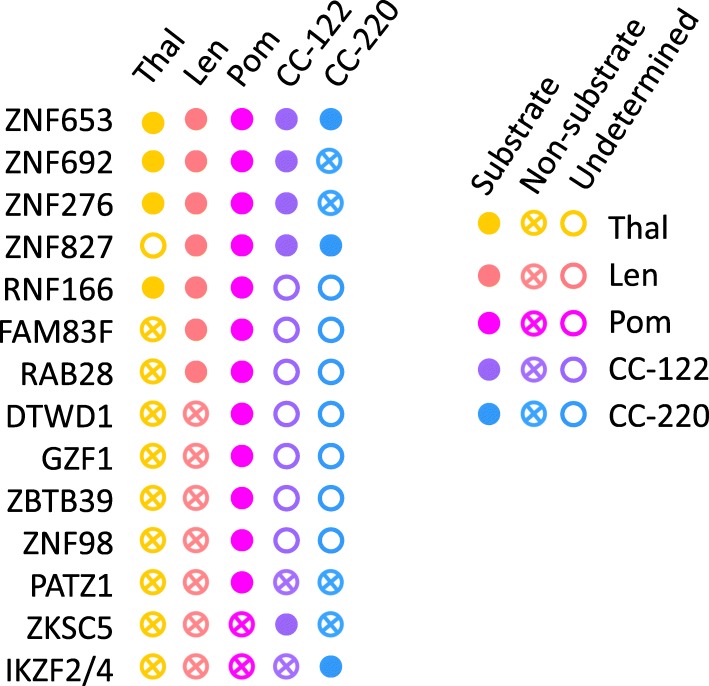
Potential neo-substrates of thalidomide, lenalidomide, pomalidomide, CC-122 and CC-220. Solid spheres represent potential neo-substrates. Spheres with crosses inside represent proteins that were not degraded by the corresponding compound, at least under the condition described in the references. Hollow spheres represent undetermined proteins. The five compounds were shown in different colors, as indicated. Thal, thalidomide. Len, lenalidomide. Pom, pomalidomide

Another study screening for C2H2 zinc finger (ZF) domains which might be targeted by IMiDs-CRL4^CRBN^ complex also discovered several neo-substrates [41]. In this study, cDNAs of 6572 C2H2 ZFs were cloned into a degradation reporter vector to generate a C2H2 ZF library. This library was transduced into HEK293T cells which were then treated with pomalidomide, lenalidomide, thalidomide or DMSO, respectively. These cells were analyzed by fluorescence-activated cell sorting (FACS) and high-throughput sequencing. The results showed that 11 ZFs were degraded by IMiDs and 6 of the 11 full length proteins can be degraded, including IKZF1/IKZF3, ZFP91, ZNF692, ZNF276, ZNF653, and ZNF827 [41]. The degradation of these transcription factors was also tested in the presence of CC-122 and CC-220. These potential neo-substrates have been summarized (Fig. 2).

IMiDs-induced substrate degradation can be affected by a series of factors, including drug concentrations, exposure time and cell types. Thus, these potential neo-substrates should be further validated under physiological or pathological conditions. IMiDs-induced degradation of target proteins, especially transcription factors previously perceived to be undruggable, provides a strategy to degrade cellular targets, which may have huge potential in the development of novel therapeutics.

## PROTAC: the ubiquitin-proteasome system-mediated degradation of target proteins

**Fig. 3:**
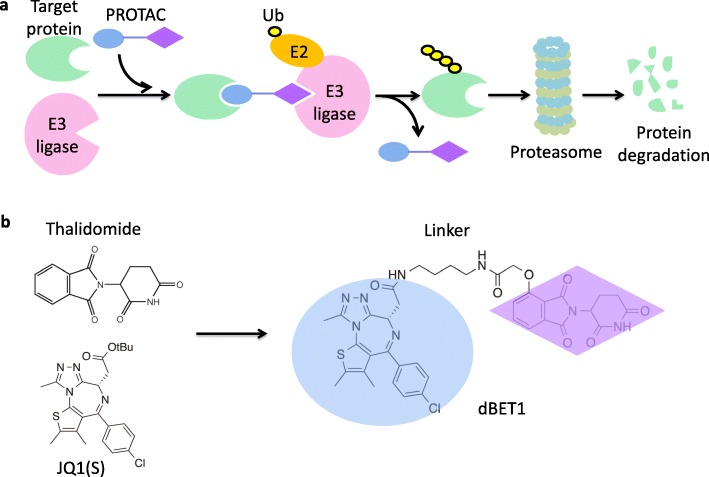
Targeting protein for degradation by Proteolysis-targeting chimeras (PROTACs). **a** PROTACs contain an E3 ligase binding moiety (purple rhombus), attached to another small molecule (blue oval) binding to the target protein through a linker. PROTACs can bring target protein to the E3 ligase for ubiquitination and subsequent degradation. **b** Chemical structure of thalidomide, JQ1(S) and one published PROTAC, dBET1. (adapted from Winter, GE, et al., Science 2015)

Proteolysis-targeting chimeras (PROTACs) are a class of bi-functional molecules designed to selectively degrade target proteins via cellular quality control machinery, such as the ubiquitin-proteasome system. Typically, these molecules contain an E3 ligase binding moiety, such as thalidomide analogs or a ligand to von Hippel-Lindau (VHL) E3 ligase, attached to another small molecule binding to a protein of interest through a linker (Fig. 3a) [53–55].

Based on this design principle, thalidomide was linked to JQ1 [56], a small molecule binding to Bromodomain-Containing Protein 4 (BRD4), to generate a bi-functional molecule called dBET1 (Fig. 3b). This PROTAC induces CRBN-dependent degradation of BRD4 and subsequent down-regulation of MYC, leading to cytotoxicity of AML cells [57]. Similar design of bi-functional degraders induce degradation of BCR-Abl [58] and Bruton’s tyrosine kinase (BTK) [59], showing therapeutic potentials in the treatment of chronic myeloid leukemia (CML) and B-cell lymphoma, respectively.

Since 2015, more and more PROTACs have been synthesized to target a broad number of cellular proteins and most of them were studied in cultured cells or animal models [57–66]. ARV-110 is an oral PROTAC that can target androgen receptor (AR) and induce AR degradation. ARV-110 is currently being tested in a phase 1 clinical trial (NCT03888612). This open-label, dose-escalation phase 1 study will evaluate the safety, tolerability, pharmacokinetics, and pharmacodynamics of ARV-110 in patients with metastatic castration-resistant prostate cancer who have progressed on at least two prior systemic therapies.

Unlike tyrosine kinase inhibitors (TKI), PROTACs do not occupy the binding site and can be recycled. Hence, in theory relatively fewer compounds can achieve expectant activities, which will make them more efficient with less off-target effects. IMiDs-based PROTACs have become a strategy of drug design for enhancing degradation of specific cellular targets. More PROTACs are expected to enter clinical development.

## Conclusions and perspectives

IMiDs are widely used clinically to treat MM, MDS with del(5q) and other hematological cancers. To achieve better efficacy, new generation of IMiDs including CC-122, CC-220, and CC-885 have been developed. CC-122 and CC-220 have entered into phase 1/2 clinical trials. Further studies are required to evaluate their efficacy in the treatment of several blood malignancies, including MM, DLBCL and NHL. CC-885 has shown potential efficacy for AML, which is not achieved by lenalidomide or pomalidomide. A number of cellular proteins have been identified to be potential neo-substrates of IMiDs, which may facilitate development of novel IMiDs. Small molecule-based PROTACs are undergoing active clinical development and are increasingly used as a new strategy for drug design. More PROTACs will be synthesized for targeted therapy. Since immunotherapy with chimeric antigen receptor (CAR)-engineered T cells becomes a highly promising therapeutic modality for cancer therapy [67–75], it is possible to combine novel IMiDs with CAR-T cells for more potent therapies of RRMM.

## Data Availability

The material supporting the conclusion of this review has been included within the article.

## Abbreviations

AML: Acute myeloid leukemia
AR: Androgen receptor
BRD4: Bromodomain-Containing Protein 4
BTK: Bruton’s tyrosine kinase
CLL: Chronic lymphocytic leukemia
CML: Chronic myeloid leukemia
CR: Complete response
CRL4: Cullin-RING Ligase 4
DLBCL: Diffuse large B-cell lymphoma
FACS: Fluorescence-activated cell sorting
IL-2: Interleukin-2
IMiDs: Immunomodulatory drugs
MCL: Mantle cell lymphoma
MDS: Myelodysplastic syndrome
MM: Multiple myeloma
MTD: Maximum tolerated dose
NHL: Non-Hodgkin’s lymphoma
NTD: Non-tolerated dose
ORR: Overall response rate
PBMCs: Peripheral blood mononuclear cells
PROTACs: Proteolysis-targeting chimeras
RRMM: Relapsed/refractory MM
SLE: Systemic lupus erythematosus
TEAEs: Treatment-emergent adverse events
TKI: Tyrosine kinase inhibitors
TMT: Tandem mass tag
VHL: Von Hippel-Lindau
